# A Population Based Study on Hepatitis B Virus in Northern Iran, Amol

**DOI:** 10.5812/hepatmon.20540

**Published:** 2014-08-17

**Authors:** Hossein Keyvani, Masoudreza Sohrabi, Farhad Zamani, Hossein Poustchi, Hossein Ashrafi, Fatemeh Saeedian, Mansooreh Mooadi, Nima Motamed, Hossein Ajdarkosh, Mahmoodreza Khonsari, Gholamreza Hemmasi, Mitra Ameli, Ali Kabir, Mahmoud Khodadost

**Affiliations:** 1 Department of Virology, Iran University of Medical Sciences, Tehran, IR Iran; 2 Gastrointestinal and Liver Diseases Research Center (GILDRC), Iran University of Medical Sciences, Tehran, IR Iran; 3 Liver and Pancreatobiliary Disease Research Center, Digestive Disease Research institute (DDRI), Tehran University of Medical Sciences, Tehran, IR Iran; 4 Department of Pathology, Kingston University, London, UK; 5 Department of Epidemiology, School of Publich Health, Shahid Beheshti University of Medical Sciences, Tehran, IR Iran

**Keywords:** Hepatitis B Virus, Infection, HBsAg, Prevalence, Epidemiology

## Abstract

**Background:**

Viral hepatitis is a major health problem worldwide. Change in transmission patterns of hepatitis B makes it necessary to re-evaluate its prevalence and risk factors.

**Objectives:**

We aimed to determine the prevalence of HBV infection and its related risk factors in Amol city, Northern Iran.

**Patients and Methods:**

As a population based study, a cluster sampling approach was used and 6146 individuals from the general population of urban and rural areas of Amol, Iran, from both genders and different ages were enrolled. Inclusion criteria were willingness to participate in the study, being a lifelong resident in Amol city or its surrounding areas with Iranian nationality. Ten milliliters (10 mL) of blood was taken from each study subject and checked regarding hepatitis B markers including HbsAg, HBsAb and HBcAb using a third generation ELISA. The prevalence of HBV infections and its potential risk factors were recorded.

**Results:**

The prevalence of HBsAg, HBsAb and HBcAb were estimated as 0.9%, 30.7% and 10.5%, respectively. The mean age of all participants was 43.9 (95% CI: 43.4, 44.3) in females and 55.6 in (n = 3472) males. In our study, there was a significant association between family history of hepatitis, rural residency and presence of HBsAg. There was also a positive correlation between HBcAb and family history of hepatitis, history of other types of hepatic diseases, history of tattooing, traditional phlebotomy, male gender and age. In backward logistic regression, a significant association was found between history of hepatitis in first-degree family members (OR = 13.35; 95% CI: 6.26, 28.47) and place of residence (OR = 2.32; 95% CI: 1.27, 4.22) with presence of HBsAg. There was also a positive correlation between history of hepatitis among first-degree family members (OR = 2.49; 95% CI: 1.52, 4.08), history of tattooing (OR = 2.13; 95% CI: 1.33, 3.42), history of previous hepatitis (OR = 1.87; 95% CI: 1.06, 3.28), male sex (OR = 1.36; 95% CI: 1.12, 1.66) and age (OR = 1.03; 95% CI: 1.03, 1.04) with presence of HBcAb.

**Conclusions:**

The prevalence of hepatitis B in Amol City and its surrounding areas was about one percent, a lower rate than other reports from Iran.

## 1. Background

It is estimated that more than 400 million individuals have hepatitis B (HBV) infection worldwide. This infection is one of the top ten leading causes of death overall (1-3). In this context, understanding regional and demographic specific prevalence of hepatitis B infection is an important issue for evaluation, national prevention programs and control of disease. A matter of greater importance is assessment of the prevalence of HBV infection regarding its transmission and demographic variation in each territory. However, global seroprevalence statistics of HBV cannot explain each region current situation, morever vaccination and other preventive policies around the world peobably make changing the epidemiological schema of HBV around the world.

The prevalence of hepatitis B varies among different countries ranging from 0.1% in Western countries to 35% in Southeast of Asia (3-5). The prevalence of hepatitis B infection in the Middle East varies among its geographic areas. While Kuwait and Bahrain have a low prevalence of hepatitis B infection of about 2%, countries like Iraq and United Arab Emirates have a prevalence of 2-5% (6, 7).

The prevalence of HBV infection in Iran varies within its geography (8). Recent data showed the overall prevalence of HBV in Iran as less than 3% (8, 9). Despite the fact, the prevalence of hepatitis B surface antigen in Iran has declined significantly during the last two decades from 3.4-8% in 1979 to 1-3% in 2000 (8, 9). So far, chronic hepatitis B remains the cause of more than 50% of cirrhosis and HCC in Iran (8, 10, 11). According to a systemic review in 2008, the seroprevalence of HBV in Iran was reported as 2.14%, although it did not reflect all provinces of Iran (12). In addition, a nationwide study performed by the Iranian Blood Transfusion Organization demonstrated that the rate of HBV infection among volunteer blood donors had decreased from 1.8% to 0.4% between 1998 and 2007 (13). A rise in general public awareness about HBV transmission along with the mass vaccination program from 1993 for all neonates and healthcare workers and those at risk of HBV infection have influenced the outcome (11, 14). Despite these studies, there is not enough data information about the epidemiology of HBV infection in various parts of Iran.

Furthermore, it seems that risk factors vary among different regions within the country; thus, determination of risk factors and establishment of the main routes of HBV transmission could in turn help health administrations design effective prevention policies.

Prevalent routes of HBV transmission include prenatal infection, sexual contact, intravenous narcotic use and transfusion. The most common route of transmission of hepatitis B in Iran was vertical transmission in the past, but other routes of transmission should also be considered. Although global vaccination program against hepatitis B decreased the rate of hepatitis B carriers, the rate of hepatitis B infected subjects remains noticeably high. This may reflect changes in patterns of epidemiology and transmission of HBV, producing some problems for healthcare systems (14-17).

## 2. Objectives

In this study, we tried to determine the prevalence and common risk factors of HBV infection in Amol city, Mazandaran province, Northern Iran.

## 3. Patients and Methods

### 3.1. Study Area and Population

Mazanderan province is located in North of Iran and Amol is a large city within this area with a population of more than 300000. There is an almost equal distribution regarding the number of its rural and urban inhabitants, providing adequate sample size for comparison between these inhabitants. Furthermore, due to a low rate of migration to and from this area, the ethnicity of the residents has to a large extent remained unchanged largely.

The study was performed as a part of a health cohort conducted in Amol from 2008 to 2011, by the Gastrointestinal and Liver Disease Research Centre (GILDRC) on the above population to determine the prevalence of HBV infection and its risk factors. Those enrolled were older than 10 years. Urban areas were considered as urbanized municipal areas and rural areas were those with agricultural areas. Inclusion criteria were Iranian nationality, lifelong residency in Amol or its vicinity, and willingness to participate in the study.

### 3.2. Sample of Study

A clustered random sampling was used, and Health Centers (HC) were considered as clusters. Each health center provided services to an average of 30 households. In this study, 24 and 16 urban and rural health centers were selected, respectively. From each health center, households were selected randomly. Then from each family, one male and one female member were chosen randomly. Finally, the total population was 6146 subjects.

### 3.3. Data Collection and Laboratory Tests

The researchers held face-to-face interviews with participants in health centers, between 2008 and 2011. When participants failed to attend their three scheduled interviews, an appointment was offered at their homes. When a subject refused to participate in this study, they were replaced other participants from their same cluster. Subjects were asked to complete a questionnaire containing information about age (number of years according to their Identification documents), gender (male or female), marital status (single or married based on the subject's own responses), family history (first degree relative: father, mother, wife/husband, siblings; second degree relatives: uncles, aunts; third degree relatives: extended family), occupation (high-risk jobs such as medical staff and nursing home workers), literacy level (five level: illiterate, < 6 years of schooling, ≤ 12 years of schooling, ≤ bachelor's degree holder, ≥ master's degree holder), blood transfusion history, history of surgery (both minor and major operation), viral hepatitis (hepatitis C, hepatitis B), narcotic drug use (usual use of narcotics or drugs more than once weekly), smoking (active smoker or passive), tattooing and piercing (presence of figures, or sign or scars), cupping and blood-letting (yes or no; based on the response of subjects to question). All those eligible to participate were referred to the Amol Research Station (ARS) in the Haraz Clinic, affiliated to the GILDRC, for completion of clinical and laboratory evaluations. HBV markers including HbsAg, HBsAb and HBcAb were evaluated using a third generation ELISA (Enzyme-Linked Immuno-Sorbent Assay) method by Acon kits (Acon lab. San Diego. CA92121, USA).

### 3.4. Statistical Analysis

Data was analyzed using STATA software, version 12 (StataCorp, Texas, USA). Since data were considered as aggregated and our sampling was clustered, we used survey data analysis methods for all computations. Each health center was considered a cluster and each participant was designated to a certain cluster. Health centers defined as ‘strata’ and the population represented by each strata used for ‘finite population correction’ using STATA ‘survey data analysis’. The data was weighted by the proportion of the population represented by each strata (health centers) to the selected subjects number of each health centers. We used survey data analysis methods to estimate more accurate HBV infection prevalence and risk factors including weighted point estimates prevalence rates of HBV biomarkers and their estimated 95% confidence intervals. ‘Proportions for survey data’ were used to calculate proportions and 95% confidence intervals. ‘Means estimate for survey data’ were used to calculate means and 95% confidence intervals. A ‘two-way table for survey data’ was used to investigate the association between HBV biomarkers and other variables. All confidence intervals, chi-square and P-values reported here were corrected for the cluster survey design using STATA ‘survey data analyses. ‘Logistic regression for survey data (backward method)’ was used to identify predictors of high-risk hepatitis B infection. All variables with p-values less than 0.20 were included in this model. A P value less than 0.05 was considered significant.

### 3.5. Ethics

Participants were informed about the project and written consent was obtained from all of them before data collection. All data was securely recorded in the study database. The study protocol and consent form were approved by the Board of Ethics of the GILDRC.

## 4. Results

### 4.1. Statistical Characteristics of Subjects

From 7000 subjects who were invited, 6146 cases (response rate = 87.8%) accepted to participate. Sixty-four subjects were excluded because of uncompleted data. Of 6146 subjects 3472 (55.6%) were male. The mean age of participants was 43.9 (95% CI: 43.4, 44.3) and 3006 (38.7%) were from rural areas. The mean age of rural participants was 43.54 (95% CI: 42.7, 44.06) and 63% (n = 1893) were male. For urban cases, these values were 44.2 (95% CI: 43.5, 44.8) and 50.9% (n = 1579), respectively. Of all participants, 286 (4.6%) individuals were younger than 18 years.

### 4.2. Prevalence of HBV Markers

Using survey data analysis method, the prevalence of HBsAg, HBsAb and HBcAb were estimated as 0.9% (95% CI: 0.6, 1.2%), 30.7% (95% CI: 29.3, 32%) and 10.5% (95% CI: 9.6, 11.4%), respectively. In total, 286 individuals were younger than 18 years, of whom 98 (37.8%) had HBsAb levels more than ten microliters. Moreover, 459 (8.27%) individuals had positive results for both HBsAb and HBcAb (Resolved infection) and 1231 (22.43%) individuals were HBsAb positive but HBcAb negative (Table 1).

**Table 1.** Prevalence of Hepatitis B Markers Among All Participants Regarding Gender and Location ^a^

HBV Markers	Total	Urban		Rural	
		Male	Female	Male	Female
**HBsAg+ ** ^**b**^	62 (0.92)	16 (0.92)	6 (0.34)	27 (1.39)	13 (1.32)
**HBsAb+ ** ^**c**^	1693 (30.73)	438 (31.79)	456 (35.12)	513 (27.26)	286 (25.99)
**HBcAb+ ** ^**d**^	639 (10.56)	190 (12.38)	128 (8.57)	213 (11.15)	108 (9.79)
**HBsAb+ and HBcAb+**	459 (8.27)	127 (9.7)	93 (6.99)	156 (8.24)	83 (7.98)
**HBsAb+ and HBcAb-**	1231 (22.43)	311 (22.11)	363 (28.12)	356 (18.99)	201 (17.85)
**HBsAb- and HBcAb+**	144 (2.26)	39 (2.61)	24 (1.62)	57 (2.9)	24 (1.72)

^a^ Data are presented as No. (%).

^b^ Hepatitis B surface antigen.

^c^ Hepatitis B surface antibody.

^d^ Hepatitis B core antibody.

### 4.3. Prevalence of HBV Risk Factors

Distribution of potential HBV risk factors between positive HBsAg and HBcAb subjects were shown in Table 2. Family history of hepatitis, rural residency and history of surgery were significantly associated with presence of HBsAg. Furthermore, there was a significant association between family history of hepatitis, family history of cirrhosis, male gender, history of traditional phlebotomy and history of tattooing with the presence of HBcAb.

**Table 2.** Distribution of HBV Risk Factors Regarding HBV Markers ^a^

Variables	HBsAg ^b^			HBcAb ^c^		
	Total	Positive	P Value ^d^	Total	Positive	P Value ^e^
**Gender**						0.001
Male	3469 (57.08)	43 (1.13)	0.09	3469 (57.10)	403 (11.84)	
Female	2608 (42.91)	19 (0.65)		2606 (42.89)	236 (8.96)	
Total	6077	62 (1.02)		6075	639 (10.51)	
**Region**						0.87
Rural	3006 (49.46)	40 (1.36)	0.01	3003 (49.43)	321 (10.64)	
Urban	3071 (50.53)	22 (0.63)		3072 (50.56)	318 (10.51)	
Total	6077	62 (1.02)		6075	639 (10.51)	
**History of surgery**						0.44
Yes	2687 (44.53)	20 (0.63)	0.04	2684 (44.65)	301 (10.79)	
No	3326 (55.31)	41 (1.14)		3327 (55.34)	324 (10.11)	
Total	6013	61 (1.01)		6011	625 (10.39)	
**History of unsterile puncture**						0.71
Yes	1290 (21.42)	10 (0.57)	0.13	1288 (21.40)	135 (10.14)	
No	4730 (78.57)	51 (1)		4730 (78.59)	493 (10.52)	
Total	6020	61 (1.01)		6018	574 (9.53)	
**History of blood transfusion**						0.06
Yes	350 (5.81)	6 (1.88)	0.12	350 (5.81)	51 (13.68)	
No	5672 (94.18)	55 (0.85)		5670 (94.18)	577 (10.24)	
Total	6022	61 (1.01)		6020	628 (10.43)	
**History of traditional phlebotomy**						0.002
Yes	460 (7.63)	6 (1.15)	0.55	460 (7.64)	70 (15.13)	
No	5561 (92.36)	55 (0.89)		5559 (92.35)	558 (10.05)	
Total	6021	61 (1.01)		6019	628 (10.43)	
**Family history of hepatitis**						< 0.001
First degree	145 (2.40)	14 (8.49)	< 0.001	145 (2.40)	37 (23.53)	
Second degree	75 (1.24)	1 (1.28)		74 (1.22)	14 (15.69)	
Third Degree	53 (0.87)	0		53 (0.88)	8 (19.83)	
No	5750 (95.46)	46 (0.72)		5749 (95.48)	569 (9.95)	
Total	6023	61 (1.01)		6021	628 (1.04)	
**History of Tattooing**						0.01
Yes	194 (3.22)	4 (1.65)	0.26	194 (3.22)	26 (16.92)	
No	5828 (96.77)	57 (0.89)		5826 (96.77)	602 (10.24)	
Total	6022	61 (1.01)		6020	628 (10.43)	
**History of imprisonment**						0.76
Yes	86 (1.42)	1 (1.09)	0.85	86 (1.42)	9 (9.47)	
No	5933 (98.57)	60 (0.91)		5931 (98.57)	619 (10.47)	
Total	6019	61 (1.01)		6017	628 (10.43)	
**High risk occupational**						0.25
Yes	58 (0.96)	0	0.50	58 (0.96)	8 (15.65)	
No	5958 (99.03)	61 (0.92)		5956 (99.03)	620 (10.4)	
Total	6016	61 (1.01)		6014	628 (10.44)	
**History of unsafe sexual contact**						0.43
Yes	26 (0.43)	0	0.66	26 (0.43)	2 (6.20)	
No	5992 (99.56)	61 (0.91)		5990 (99.56)	626 (10.47)	
Total	6018	61 (1.01)		6016	628 (10.43)	
**History of IDU ** ^**f**^						0.10
Yes	27 (0.44)	0	0.64	27 (0.44)	0	
No	5994 (99.55)	61 (0.91)		5992 (99.55)	627 (10.48)	
Total	6021	61 (1.01)		6019	627 (10.41)	

^a^ Data are presented as No. (%).

^b^ Hepatitis B surface antigen.

^c^ Hepatitis B core antibody.

^d^ P-value for HBV risk factors regarding HBsAg.

^e^ P-value for HBV risk factors regarding HBcAb.

^f^ Intravenous drug user.

### 4.4. Association Between HBV Markers and its Risk Factors

Logistic regression analysis was used to calculate the odds ratios for main risk factors (Tables 3 and 4). In univariate logistic regression (model 1), there was a significant association between history of hepatitis in family members (OR = 12.77), rural region residents (OR = 2.15), and the presence of HBsAg. There was also a positive correlation between HBcAb and history of hepatitis in family members (OR = 2.7), history of other types of hepatic diseases (OR = 1.9), history of tattooing (OR = 1.7), traditional phlebotomy (OR = 1.5), male sex (OR = 1.3) and age (OR = 1.03).

**Table 3.** Logistic Regression for Assessing Association Between HbsAg and Related Risk Factors

Variables	OR (95% CI) Model 1 ^a^	OR (95% CI) Model 2 ^b^	OR (95% CI) Model 3 ^c^	OR (95% CI) Model 4 ^d^
**Gender**				
Male	1.72 (0.90 - 3.27)	1.72 (0 .90 - 3.27)	1.33 (0 .52 - 3.35)	-
Female	Reference	Reference	Reference	-
**Age, y**	1.00 (0.99 - 1.02)	1.00 ( 0.99 - 1.02)	-	-
**Region**				
Rural	2.15 (1.18 - 3.92)	2.05 ( 1.10 - 3.83)	2.32 ( 1.23 - 4.37)	2.32 (1.27 - 4.22)
Urban	Reference	Reference	Reference	Reference
**Family history of hepatitis**				
First degree	12.77 (6 - 27.19)	13.68 (6.36 - 29.44)	12.60 (5.88 - 27.03)	13.35 (6.26 - 28.47)
Second degree	1.79 (0.24 - 13.09)	1.88 (0.25 - 13.73)	1.54 (0.23 - 9.98)	1.73 (0.23 - 12.56)
Third degree	0.29 (0.04 - 1.91)	0.35 (0.05 - 2.26)	0.38 (0.05 - 2.47)	0.33 (0.05 - 2.11)
No	Reference	Reference	Reference	Reference
**History of blood transfusion**	2.22 (0.78 - 6.34)	2.21(0.74 - 6.63)	2.35 (0.81 - 6.76)	-
**History of surgery**	0.55 (0.30 - 0.99)	0.56 (0.31 - 1.03)	0.55 (0.28 - 1.09)	-
**History of imprisonment**	1.20 (0.16 - 8.73)	0.92 (0.12 - 6.78)	-	-
**History of IDU ** ^**e**^	1.74 ( 0.10 - 28.37)	-	-	-
**History of tattooing**	1.87 (0.60 - 5.78)	1.89 (0.61 - 5.83)	1.87 (0.65 - 5.37)	-
**History of traditional phlebotomy**	1.29 (0.54 - 3.12)	1.18 (0.46 - 2.99)	-	-
**History of unsterile puncture**	0.56 (0.27 - 1.18)	0.78 (0.29 - 2.08)	0.70 (0.25 - 1.90)	-
**High risk occupation**	0.89 (0.05 – 14.32)	-	-	-
**History of Alcohol addiction**	1.36 ( 0.41 - 4.53)	1.12 (0.33 - 3.83)	-	-
**History of other **types** of **h**epatitis**	1.99 (0.76 - 5.18)	1.97 (0.74 - 5.23)	1.63 (0.39 - 6.78)	-
**History of unsafe sexual contact**	1.80 (0.11 - 29.46)	-	-	-

^a^ Crude Odds Ratio.

^b^ Adjusted Odds Ratio for sex and age.

^c^ Adjusted Odds Ratio (all variables with p-value < 0.2 entered the model such as sex, region, family history of hepatitis and history of blood transfusion, surgery, tattooing, unsterile puncture, icterus and other types of hepatitis).

^d^ Multivariable backward logistic regression model.

^e^ Intravenous drug user.

**Table 4.** Logistic Regression for Assessing Association Between HBcAb and Related Risk Factors

Variables	Model 1, OR (95% CI) ^a^	Model 2, OR (95% CI) ^b^	Model 3, OR (95% CI) ^c^	Model 4, OR (95% CI) ^d^
**Gender**				
Male	1.36 (1.12 - 1.64)	1.03 (1.03 - 1.04)	1.37 (1.12 - 1.67)	1.36 (1.12 - 1.66)
Female	Reference	Reference	Reference	Reference
**Age, y**	1.03 ( 1.03 - 1.04)	1.34 (1.11 - 1.63)	1.03 (1.03 - 1.04)	1.03 (1.03 - 1.04)
**Region **				
Rural	1.01 (0.84 - 1.21)	0.99 (0.82 - 1.20)	-	-
Urban	Reference	Reference	-	-
**Family history of hepatitis**				
First degree	2.78 (1.81 - 4.26)	2.13 (1.98 - 4.97)	2.40 (1.45 - 3.97)	2.49 (1.52 - 4.08)
Second degree	1.68 (0.88 - 3.21)	1.77 (0.91 - 3.42)	1.31 (0.69 - 2.46)	1.39 (0.74 - 2.61)
Third degree	2.23 (0.94 - 5.29)	2.76 (1.06 - 7.13)	2.50 (0.97 - 6.43)	2.50 (0.97 - 6.43)
No	Reference	Reference	Reference	Reference
**History of blood transfusion**	1.38 (0.97 - 1.97)	1.17 (0.81 - 1.70)	-	-
**History of surgery**	1.07 (0.89 - 1.29)	0.92 (0.76 - 1.12)	-	-
**History of imprisonment**	0.89 (0.43 - 1.82)	0.64 (0.31 - 1.34)	-	-
**History of IDU ** ^**e**^	1.42 (0.009 - 2.51)	-	-	-
**History of tattooing**	1.78 (1.11 - 2.84)	2.19 (1.36 - 3.52)	2.14 (1.33 - 3.43)	2.13 (1.33 - 3.42)
**History of traditional Phlebotomy**	1.59 (1.17 - 2.15)	1.10 (0.81 - 1.50)	1.06 (0.77 - 1.45)	-
**History of unsterile puncture**	0.95 (0.76 - 1.19)	1.05 (0.79 - 1.40)	-	-
**High risk occupation**	1.59 (0.70 - 3.59)	1.79 (0.79 - 4.07)	-	-
**History of Alcohol addiction**	1.12 (0.67 - 1.88)	1.21 (0.71 - 2.05)	-	-
**History of other type**s** of **h**epatitis**	1.99 (1.07 - 3.72)	1.95 (1.14 –3.33)	1.84 (1.04 - 3.25)	1.87 (1.06 - 3.28)
**History of unsafe sexual contact**	0.56 (0.13 - 2.40)	0.65 (0.15 - 2.88)	-	-

^a^ Crude Odds Ratio.

^b^ Adjusted Odds Ratio for sex and age.

^c^ Adjusted Odds Ratio (all variables with p-value < 0.2 entered the model such as sex, age, family history of hepatitis, family history of cirrhosis and history of tattooing, traditional phlebotomy, icterus and other types of hepatitis).

^d^ Multivariable backward logistic regression model.

^e^ Intravenous drug user.

In multivariable logistic regression, after adjustment for demographic variables such as age and sex (Model 2), there was a significant association between family history of hepatitis (OR = 13.6), and rural residency (OR = 2) with the presence of HBsAg. There was also a positive correlation between HBcAb and history of tattooing (OR = 2.1), family history of hepatitis B (OR = 2.1) and history of other types of hepatic diseases (OR = 1.95) in a similar model adjusted for age and sex.

In model 3, all variables with p-values less than 0.2 included in the model. There was a significant association between history of hepatitis in family members (OR = 12.6) and rural region (OR = 2.3) with the presence of HBsAg. Finally, in the backward method of multivariable logistic regression (model 4), we found a significant association between history of hepatitis in first relative members (OR = 13.3) and region (OR = 2.3) with the presence of HBsAg. A history of hepatitis in first-degree relatives (OR = 2.4), history of tattooing (OR = 2.1), history of other types of hepatitis disease (OR = 1.8), male sex (OR = 1.3) and age (OR = 1.03) with the presence of HBcAb in this model (Tables 3 and 4).

## 5. Discussion

In this survey, serum samples of 6082 subjects were evaluated for the presence of HBsAg and HBcAb. According to our survey analyses, the prevalence of HBsAg and HBcAb were 0.9% and 10.5%, respectively.

In our study, the prevalence of HbsAg as an indicator of chronic hepatitis B infection was lower than other parts of Iran and lower than the national prevalence rate. Zali et al. and Alavian et al. revealed a prevalence of 1.7% and 2.14% for HBsAg in the general population of Iran (8, 12), indicating that the rate of HBV infection does not follow a consistent pattern in various geographical areas within the country. Table 5 summarizes some population based studies published on the prevalence of HBsAg in Iran. According to these studies, the highest prevalence of HBV infection was in Golestan province (18) northeast of Iran and the lowest rate of HBV infection in Azarbayjan province (12) northwest of Iran. Although, the frequency of hepatitis B was significantly different in Amol located between these two areas and adjacent to Golestan province (0.9% vs. 5.1%). We could not express a clear explanation about these differences, but it might be due to lifestyle, population density, interfamilial contact, public education about HBV infection and successful anti HBV vaccination. Nationwide vaccination against HBV was introduced in Iran since 1992; therefore, subjects younger than 18 years had received vaccination against HBV. In this population, lower expected number of subjects with positive HBsAb (37.8%) might be due to the decreasing level of antiHBs during the time after vaccination.

HBcAb is another marker of viral hepatitis B and an indicator of past exposure to HBV. Positive results for HBcAb could be considered as a potential threat for transmission of hepatitis B infection (19, 20). Moreover, a noticeable proportion of population with positive HBcAb has occult HBV infection with undetectable HBV- DNA and negative results for serum HBsAg. In this situation, further follow-up and careful evaluation of patients is needed. However, the prevalence of this HBV marker is not similar in different geographic areas of Iran. As illustrated in Table 5, our results showed a lower prevalence of HBcAb compared to other parts of Iran (12, 18, 19, 21-24). It seems that cultural factors and lack of accurate implementation of health guidelines might affect the distribution of HBV infection. The differences between prevalence of hepatitis B markers in different parts of Iran may be related to some traditional lifestyle factors, population density, public knowledge and education about HBV transmission. Mass vaccination program and active follow-up of patients with HBV infection by health service providers had a significant influence on the rate of infection. However, it is not an easy task to compare different prevalence rates among provinces of Iran.

**Table 5.** The Prevalence of HbsAg and HBcAb in Various Provinces of Iran

Province	HBcAb	HbsAg	Author
**Sistan Balochestan**	23.58	3.38	Salehi et al. (2012) (21)
**Golestan**	36.9	5.1	Merat et al. (2009) (18)
**Hormozgan**	13.3	2.7	Merat et al. (2009) (18)
**Tehran**	14.2	2.3	Merat et al. (2009) (18)
**Khorasan-e-Razavi**	-	1.39	Fathimoghaddam et al. (2011) (23)
**Qom**	-	1.3	Ghadir et al. (2012) (24)
**Azarbayjan**	-	1.2	Alavian et al. (2008) (12)
**Mazandaran**	10.5	1	Present study
**Fars**	6.55	-	Behbahani et al. (2006) (22)

Multivariate analysis showed that HBsAg positivity was significantly correlated with family history of hepatitis and rural residency as independent risk factors. Our results revealed that all age groups and particularly males had exposure to HBV. Furthermore, family history, tattooing, male gender, age of hepatitis and tattooing had significant correlations with the presence of HBcAb (Tables 3 and 4).

**Figure 1.**
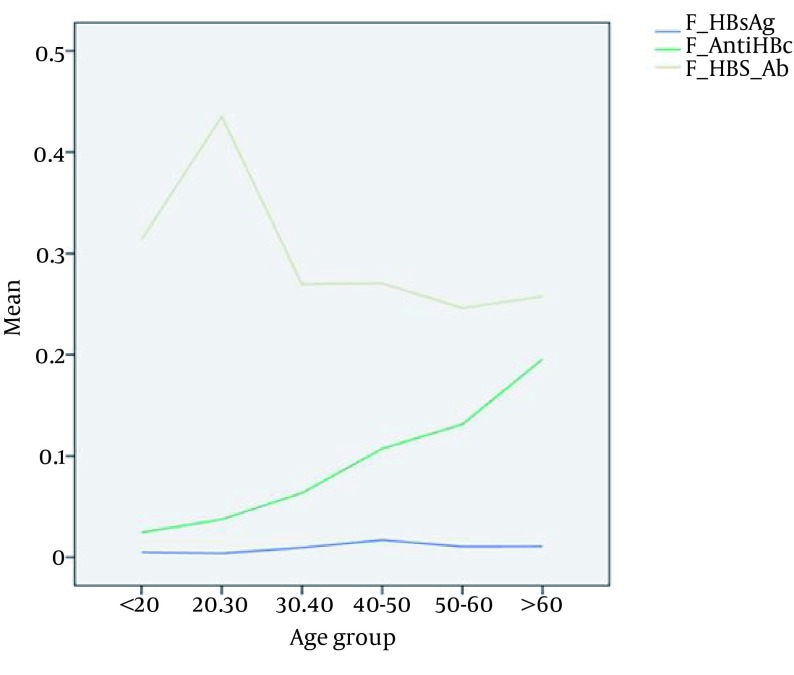
The Prevalence of HBV Markers Related to Age Groups

In almost all previous studies in Iran, age was considered a predictive risk factor in accordance with our study (Figure 1) (17, 18, 23, 25). This fact could be attributed to the mass national vaccination program in Iran from 1992 covering the young population; therefore, older populations had not received vaccination against HBV and are more prone to HBV infection through various other ways.

In concordance with other published reports, our findings showed that HBV infection is more common in males than females (12, 18, 23). Although in the forward regression analysis of our study, there was an association between HBsAg and gender, while the backward regression analysis did not show the same result. Alavian et al. revealed a higher prevalence of HBV among men than women in Iran (2.55% vs. 2.03% respectively) (12). Some research groups did not find a difference between genders (9, 18, 23, 25). This difference could be related to higher exposure of males to hepatitis B risk factors.

In a study on 1264 HBV positive subjects, it was concluded that family history of hepatitis, history of dental surgery, history of transfusion and marriage were risk factors of infection. Therefore, marriage status can be considered as an interfamilial transmission in adults who have not received vaccination (23, 26).

There are a limited number of studies comparing the prevalence of HBV infection in rural and urban areas in Iran. In our study, the prevalence of HBsAg in rural areas was twice as high as urban areas (1.36% vs. 0.63%). This result is comparable with the findings of other investigators in different provinces of Iran (25). However, the rates of HBcAb in urban and rural areas in the current study were quite similar (10.51% and 10.64%, respectively).

In conclusion, the prevalence of HBV infection in the north central province, northern Iran was almost lower than other reports from Iran country. Moreover, continuing public education about hepatitis B as well as adequate vaccination and screening programs among high-risk groups should be considered to control the infection.
